# Development and validation of an rDNA operon based primer walking strategy applicable to *de novo* bacterial genome finishing

**DOI:** 10.3389/fmicb.2014.00769

**Published:** 2015-01-21

**Authors:** Alexander W. Eastman, Ze-Chun Yuan

**Affiliations:** ^1^Southern Crop Protection and Food Research Centre, Agriculture and Agri-Food Canada, Government of CanadaLondon, ON, Canada; ^2^Department of Microbiology and Immunology, Schulich School of Medicine and Dentistry, University of Western OntarioLondon, ON, Canada

**Keywords:** genome finishing, ribosomal DNA, contigs assembly, bacterial genomics, second-generation sequencing (SGS)

## Abstract

Advances in sequencing technology have drastically increased the depth and feasibility of bacterial genome sequencing. However, little information is available that details the specific techniques and procedures employed during genome sequencing despite the large numbers of published genomes. Shotgun approaches employed by second-generation sequencing platforms has necessitated the development of robust bioinformatics tools for *in silico* assembly, and complete assembly is limited by the presence of repetitive DNA sequences and multi-copy operons. Typically, re-sequencing with multiple platforms and laborious, targeted Sanger sequencing are employed to finish a draft bacterial genome. Here we describe a novel strategy based on the identification and targeted sequencing of repetitive rDNA operons to expedite bacterial genome assembly and finishing. Our strategy was validated by finishing the genome of *Paenibacillus polymyxa* strain CR1, a bacterium with potential in sustainable agriculture and bio-based processes. An analysis of the 38 contigs contained in the *P. polymyxa* strain CR1 draft genome revealed 12 repetitive rDNA operons with varied intragenic and flanking regions of variable length, unanimously located at contig boundaries and within contig gaps. These highly similar but not identical rDNA operons were experimentally verified and sequenced simultaneously with multiple, specially designed primer sets. This approach also identified and corrected significant sequence rearrangement generated during the initial *in silico* assembly of sequencing reads. Our approach reduces the required effort associated with blind primer walking for contig assembly, increasing both the speed and feasibility of genome finishing. Our study further reinforces the notion that repetitive DNA elements are major limiting factors for genome finishing. Moreover, we provided a step-by-step workflow for genome finishing, which may guide future bacterial genome finishing projects.

## Introduction

The advent of high-throughput second-generation sequencing platforms has allowed for a monumental leap in the affordability and feasibility of genomic studies (Bentley, 2010; Delseny et al., 2010). Applications of these second-generation sequencing technologies have been especially helpful and disruptive during the characterization of microorganisms, where whole genome sequencing has become a routine step in bacterial strain characterizations (Medini et al., 2008; Maclean et al., 2009).

Four second-generation sequencing platforms are commonly employed for *de novo* sequencing of bacterial genomes; Illumina, 454 Roche, SOLiD and Ion Torrent. With the exception of SOLiD, second-generation sequencing technologies determine the sequence of short, fragmented DNA molecules through detection of nucleotides incorporated during synthesis of a complementary strand. Each technology varies in the specifics of the sequencing chemistry, read-length, library preparation and output accuracy, and the advantages and disadvantages of each specific technology have been reviewed previously (Miller et al., 2008; Glenn, 2011; Quail et al., 2012; McGinn and Gut, 2013; Morey et al., 2013).

In second-generation sequencing platforms, sequencing reads are assembled by computational assignment of nucleotide identities. Where applicable, software trims the known adaptor sequences from the DNA sequences, and removes bases and reads that do not meet quality thresholds and *in silico* assembly algorithms are used to assemble the remaining reads into contiguous sequences (contigs) (Pop et al., 2004; Horner et al., 2009; Gritsenko et al., 2012). Detailed methods using the power of second-generation sequencing platforms for various experimental aims are available; however there are few examples in the literature describing the finishing procedures for bacterial genomes. *De novo* assembly of a bacterial genome results in multiple contigs ranging in length from a few hundred to thousands of base pairs, commonly referred to as a draft genome (Tsai et al., 2010; Wetzel et al., 2011). Taken alone, contigs contained within a draft genome offer no spatial orientation relative to each other and are numbered in descending order by size. In addition, draft genomes are error-prone, containing frame shifts, missing genes/sequence, rearrangements, ambiguous bases, and sequencing artifacts and do not always accurately reflect genome structure (Poptsova and Gogarten, 2010; Ricker et al., 2012). The increased accuracy and fidelity of completely sequenced and finished genomes are preferential to draft genomes for later use in systems biology and the various “omics” fields.

*Paenibacillus polymyxa*, the type species of the *Paenibacillus* genus, have been isolated from diverse environments including marine sediments, hot-springs and soils world-wide and are commonly found in association with plant hosts. *P. polymyxa* strains are best known for promoting plant fitness through pathogen antagonism, nutrient cycling, and priming of plant defenses (Lal and Tabacchioni, 2009). Recently, interest for potential applications in agriculture and renewable bio-fuel production has driven the resurgence in studies of *P. polymyxa* biology (Yu et al., 2011; Eastman et al., 2014b). Besides our sequencing of *P. polymyxa* strain CR1 (here forth, CR1), complete genomes of four other *P. polymyxa* strains (E681, SQR-21, M1, and SC2) are publically available among numerous draft sequences (Kim et al., 2010; Ma et al., 2011; Niu et al., 2011; Eastman et al., 2014a; Li et al., 2014).

Here we detail the sequencing, assembly, and finishing of the CR1 genome using a modified primer walking strategy developed to minimize efforts for gap closure. Our approach simplifies the sequencing procedure for repetitive rDNA operons, thereby speeding up genome sequencing, assembly, and finishing. Furthermore, we illustrate the most common approach to bacterial genome sequencing and offer an overview of the challenges of bacterial genome finishing.

## Methods and results

### Second-generation sequencing

**Figure 1:**
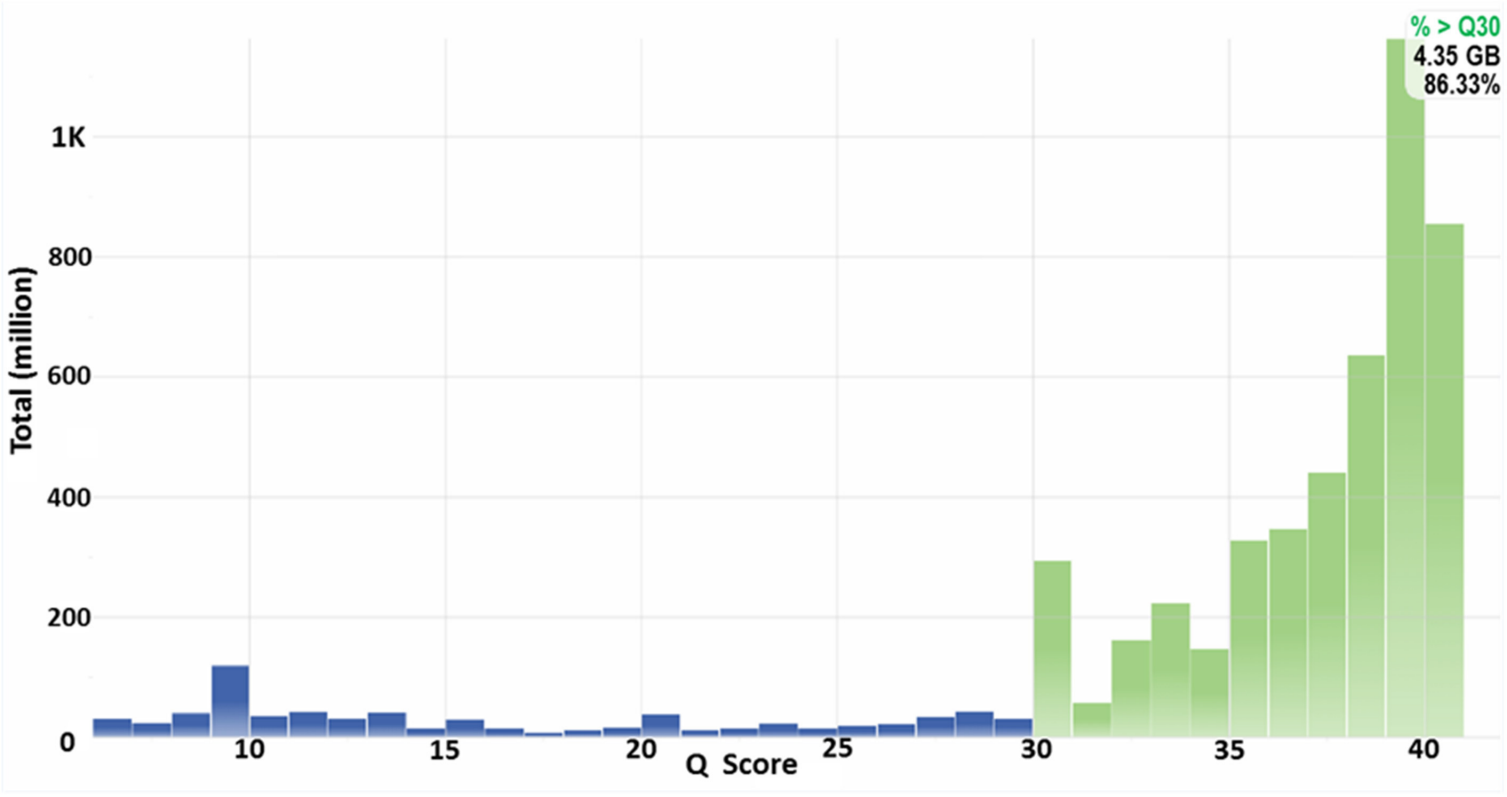
**Q_30_ graph of Illumina reads**. Phred-like quality scores (Q-scores) are used to measure the accuracy of nucleotide identity data from a sequencing run. A higher quality score indicates a lower probability that an individual base is called incorrectly, where Q = −10_log_10 (e). Q_20_ indicates the probability of an incorrect base call is 1 in 100; Q_30_, 1 in 1000; Q_40_, 1 in 10,000. The green proportion of the graph represents bases with a high quality score (defined as Q_30_) identified during the run. The whole genome *de novo* sequencing of *Paenibacillus polymyxa* strain CR1 had 86.33% = Q_30_ bases.

Whole genome sequencing using second-generation sequencing technologies is a powerful tool for microbiologists looking to identify the potential genetic basis of observed phenotypes. Prior to proceeding with completing a draft bacterial genome, the quality of the sequencing run and draft assembly must be taken into careful consideration. Adequate genome coverage of the sequencing read libraries are critical for assembling a genome with high fidelity. Typically, 40~50× coverage is needed for genome sequencing and higher coverage (>50×) does not necessarily increase assembly strength. If the sequencing depth is low it is advisable to re-sequence the genome since the power of second-generation sequencing highly relies on high coverage to overpower the limitations of short read lengths and comparatively low individual base accuracy. Currently the Q_30_ score is the *de facto* standard for measuring the accuracy of second-generation reads, a Q_30_ score represents a 1/1000 chance of an incorrect base identification (Ewing and Green, 1998; Ewing et al., 1998). It is important to note that Q_30_ is presented as a percentage of total bases in the sequencing run that reach the threshold and typically, Q_30_ scores for a high-quality run are >80%. In the case of the CR1 genome, sequencing was performed using the Illumina MiSeq platform. A short-insert read library was generated with a target insert size of 400 bp using the NexteraXT DNA sample preparation kit and a mate-pair library was generated using the Nextera mate-pair sample preparation kit. MiSeq 2× 150 bp sequencing yielded a 140× genome coverage short-insert read library (2.9 million reads) and a 107× genome coverage mate pair read library (2.2 million reads). Adaptor sequences were removed, mate-pair reads with insert sizes >400 base pairs were filtered and the two libraries were merged generating a 40× genome coverage merged library with a mean mate-pair insert size of 1.25 kb and a Q_30_ score of 86% (Figure 1).

### Genome draft assembly of *P. polymyxa* strain CR1

Concomitant with the advances of second-generation sequencing, developments of *in silico* assembly programs has yielded numerous bioinformatics tools designed to minimize and compensate for sequencing errors inherent to each respective assembly platform. Each algorithm varies the length of matching sequence required before two reads are considered overlapping (called the k-mer length), which has a dramatic effect on the generated contig assembly. A short k-mer length results in a more contiguous assembly at the cost of accuracy and fidelity, while a long k-mer length results in a highly fragmented but more accurate draft assembly (Gibbons et al., 2009). Recent tools have been developed to integrate the outputs from multiple assembly programs each with different k-mer lengths into a single set of contigs with higher strength and accuracy. The advantage of integrating the contigs generated by multiple programmes stems from the ability to combine the highly accurate, long k-mer assemblies with the less accurate but more contiguous short k-mer assemblies. In the case of CR1 genome sequencing, three separate contig assembly programs, ABySS with a k-mer length of 67 bp (Birol et al., 2009), Velvet with a k-mer length of 31 bp (Zerbino and Birney, 2008), and SOAPdenovo with a k-mer length of 55 bp (Luo et al., 2012), were run independently, generating three separate contig assemblies that are then integrated into a final draft assembly by CISA (Lin and Liao, 2013). The draft assembly of the CR1 genome contained 38 contigs with an N_50_ value of 1.5 Mb (*N*_50_ = the size of contig where 50% of the total bases are accounted for when combined with all longer contigs), representing an excellent draft genome for finishing. It may be wise to re-sequence the genome prior to finishing if the sequencing read library has poor genome coverage, a low Q_30_-score or the draft genome is highly fragmented (>100 contigs), especially in cases where no closely related strains or species are available as reference genomes for scaffold assembly.

**Figure 2:**
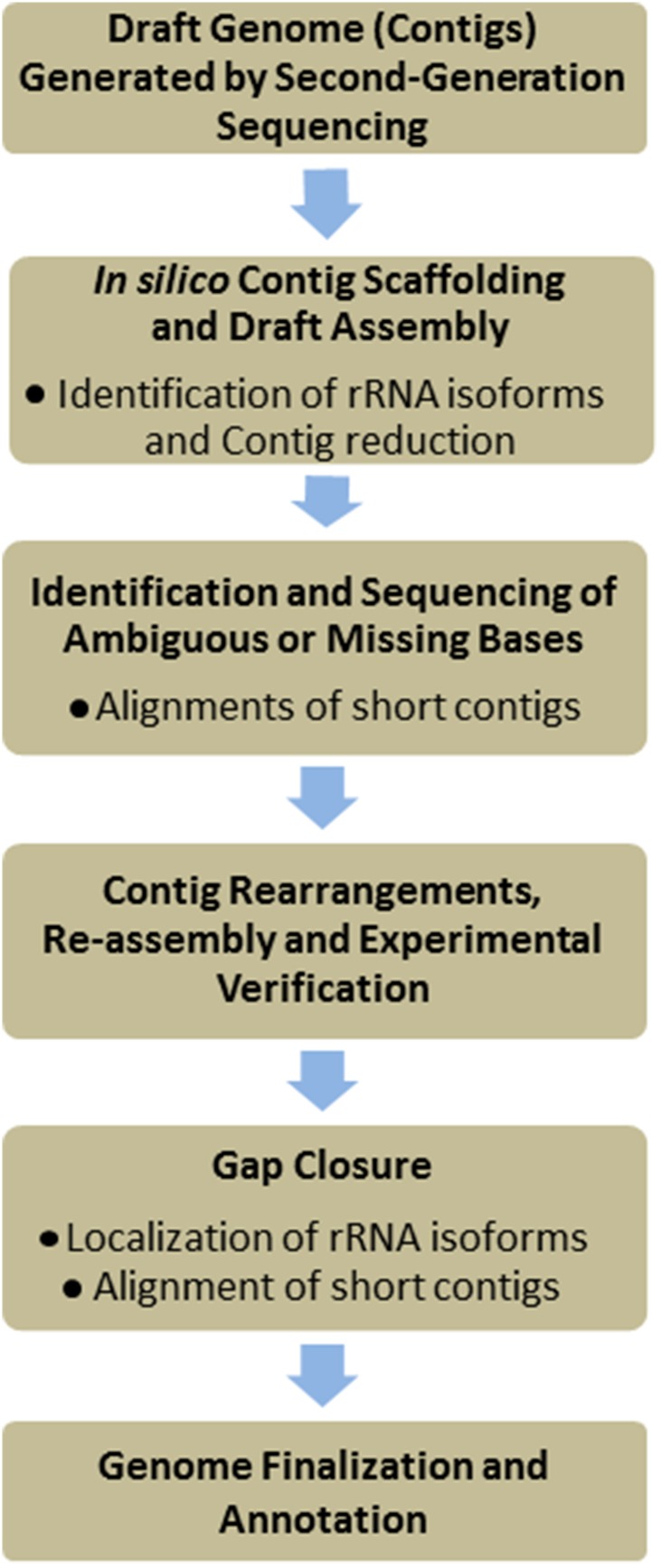
**Flow chart for *de novo* bacterial genome finishing**. The assembly process is initially simplified by removal of contigs comprised entirely of rRNA genes. The draft genome is then scaffolded against other closely related strains and species to identify potential gaps, rearrangements, and missing sequences. Ambiguities in structure and nucleotide identity are targeted for Sanger re-sequencing. Furthermore, large scale rearrangements are carefully investigated through a combination of PCR and sequencing to ensure mistakes are not incorporated during draft genome assembly. Finally gaps between contigs are sequenced and the genome is assembled and annotated.

Regardless of which technology is used to generate the draft genomic data, the procedure for genome assembly and finishing are more or less analogous (Nagarajan et al., 2010). Unfortunately, no automated/high-throughput and universally applicable method has been developed for finishing of *de novo* bacterial genomes and Sanger sequencing remains the most commonly used approach (Schuster, 2008; Hurt et al., 2012). Finishing of bacterial genomes typically involves five major steps after the assembly of a draft genome (Figure 2); locating and sequencing of ambiguous bases, scaffold assembly of contigs, investigation of contig rearrangements, sequencing of contig gaps, and genome annotation.

### Reduction of rRNA contigs by *in silico* alignment

Repetitive genetic elements are known to be causative of misassembled contigs during *de novo* genome assembly (Ricker et al., 2012). Bacterial genomes encode multiple, highly-similar operons encoding 16S, 23S, and 5S ribosomal subunits, commonly referred to as rDNA operons (Rainey et al., 1996). Manual inspection of contig ends determined that various short contigs (<2 kb) corresponded to isoforms of rRNA genes. Contigs comprised of multi-copy repetitive elements such as rDNA have the potential to be assembled from sequencing reads corresponding to distant genomic loci and are thus prone to being chimeric. We employed Sanger sequencing with universal 16S and 23S rDNA primers (Table 1) to generate consensus sequences for both the 16S and 23S genes (Anzai et al., 2000; Hunt et al., 2006). Alignment of the consensus sequences against the 38 contigs from the CR1 draft genome allowed for masking of 10 short contigs (<2 kb) that corresponded to various isoforms of rRNA genes. Thus, assembly and masking of the short contigs containing highly similar rDNA into consensus 16S and 23S genes reduced the set of contigs from 38 to 28. Within this set of 28 contigs, 24 of the remaining ends (of a possible 56 ends) contained some fragment of 5S, 16S, or 23S rRNA genes.

**Table 1:** **Universal 16S and 23S primers for rapid rRNA operon gap sequencing**.

**Primer name**	**Sequence**	**Gene**	**Direction**	**References**
8F	AGAGTTTGATCCTGGCTCAG	16S	Forward	Anzai et al., 2000
1492R	CGTTACCTTGTTACGACTT	16S	Reverse	Anzai et al., 2000
127F	CYGAATGGGRVAACC	23S	Forward	Hunt et al., 2006
2241R	ACCGCCCCAGTHAAACT	23S	Reverse	Hunt et al., 2006
U1	TGGGATACCACCCTGATCGT	16S	Reverse	This study
U2	GTTTGGGCTAATCCGCGTTC	16S	Forward	This study
U3	CCGTCACACCACGAGAGTTT	23S	Reverse	This study
U4	GTCCGCCGCTAGGTTGATTA	23S	Forward	This study

Primers are listed 5′–3′. Nucleotides are listed using IUPAC convention where; Y = C or T, R = A or G, V = A, C, or G, and H = A, C, or T. 8F/1492R and 127F/2241R are as previously described by Anzai et al. (2000) and Hunt et al. (2006), respectively. Primers U1, U2, U3, and U4 hybridize to rDNA operons as depicted in **Figure 5**.

### Scaffolding of draft genome

**Figure 3:**
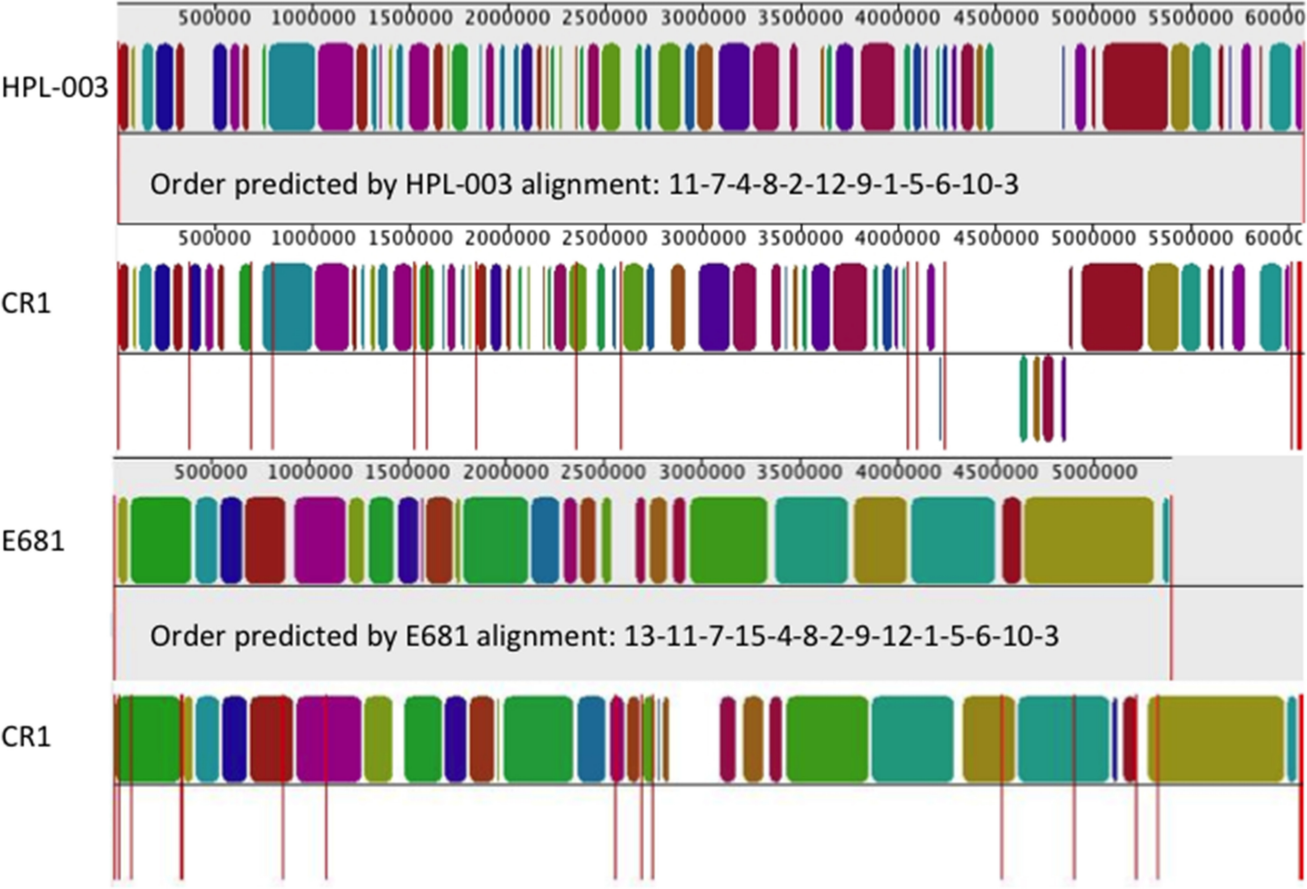
**CR1 draft genome scaffold assemblies with related reference genomes**. The local collinear block (LCB) plot was generated with the contig reordering tool within Mauve using default parameters. The name of the strain represented is listed to the left of each LCB plot. HPL-003 represents *Paenibacillus terrae* strain HPL-003, a closely related species to *P. polymyxa*. Conversely, E681 represents *P. polymyxa* strain E681, the closest related *P. polymyxa* strain to CR1. The contigs contained within the *P. polymyxa* strain CR1 draft genome are reordered to approximate the LCB plot of the above completely finished genome. Global alignments are visualized as LCBs, which represent regions with high levels of nucleotide similarity between genomes. LCBs are colored according to homology to LCBs of the compared genome. LCBs drawn below the horizontal correspond to inversions relative to the reference genome.

The explosion of second-generation sequencing projects has dramatically increased the number of published genomes from all walks of life, especially prokaryotes. Accordingly the availability of a closely related sequence facilitates scaffolding of newly obtained draft genomes against a previously completed, closely related genome, identifying putative gaps and variability in the sequence for experimental investigation. The multiple genome alignment software Mauve iteratively compares segments of DNA to user-provided genomes by characterizing regions of high similarity as local collinear blocks, which can then be used to reorder the contigs to generate the best assembly compared to any user-provided reference genome (commonly referred to as scaffolding) (Darling et al., 2010, 2011). In the case of the CR1 genome assembly, the draft genome with 28 remaining contigs was aligned using progressiveMauve against the completely sequenced genome of *P. polymyxa* strain E681 which showed the highest nucleotide-level similarity to CR1 (Kim et al., 2010). With the reference genome as scaffold, we were able to draft the final CR1 genome assembly and identify putative contig gaps for further sequencing and closure. For *de novo* sequencing projects where the bacterium is the first member of its species to be sequenced, the next closest related species may be used for scaffold assembly. For example, alignments of the CR1 draft genome against the complete *Paenibacillus terrae* strain HPL-003 genome (Shin et al., 2012) also resulted in a contig assembly that approximated the alignment against *P. polymyxa* strain E681 (Figure 3).

### Targeted sequencing of ambiguous bases

**Figure 4:**
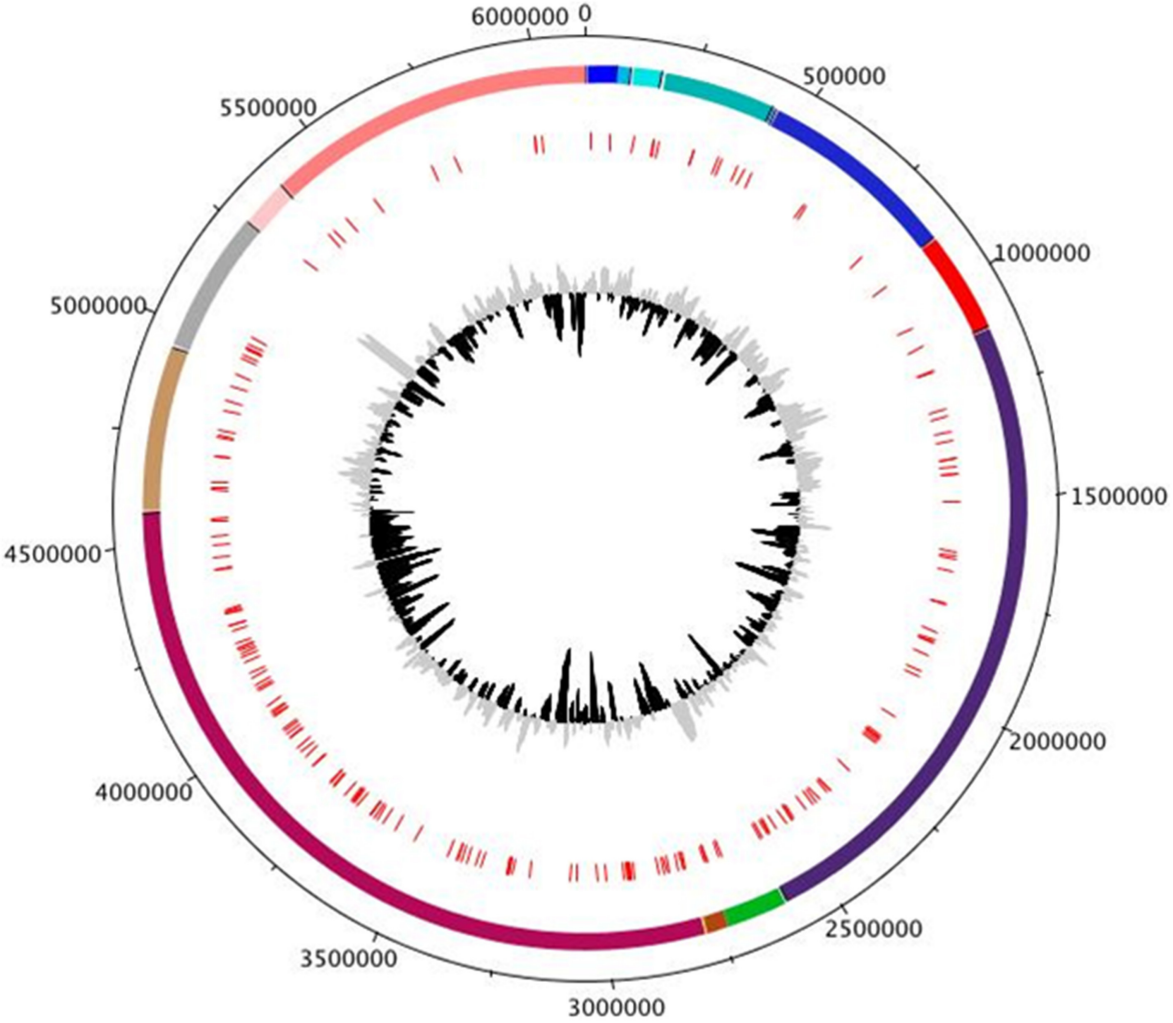
**Circular representation of CR1 scaffold assembly showing locations of ambiguous bases**. From inside to outside; 1st ring – G+C mol% content, 2nd ring – locations of ambiguous bases (red), 3rd ring – final assembly of draft contigs where separate contig from the draft assembly are labeled with different colors, 4th ring – genome size in base pairs. Annotations were performed in Artemis and visualized using DNAplotter.

Reduction of a highly fragmented draft genome comprised of numerous contigs to a single contiguous sequence is the most noticeable feature of finished genomes (Mardis et al., 2002). However, genomes sequenced using paired-end runs and assembled using Velvet or ABySS occasionally include series of ambiguous bases within the assembled contigs, represented by N nucleotides in FASTA formatted data (Mardis et al., 2002; Zerbino and Birney, 2008; Birol et al., 2009). The lengths of ambiguous base stretches can range from three to hundreds of bases in length and are determined computationally during read assembly. These ambiguous base stretches can result from mate-pair reads without sufficient internal coverage, nucleotide polymorphisms and individual bases within reads that do not meet quality thresholds. Nevertheless, ambiguous bases should be targeted for Sanger sequencing to fill in the missing bases and can be treated as short, pre-defined contig gaps during sequencing. The draft genome (28 contigs) of CR1 contained a total of 198 ambiguous base stretches, between 9 and 769 bp in length, distributed irregularly throughout the genome (Figure 4). The generated draft assembly and locations of ambiguous bases were annotated in Artemis (Rutherford et al., 2000) and visualized using DNAplotter (Carver et al., 2009). To identify ambiguous bases, 198 sets of primers flanking the 5′ and 3′ ends of each ambiguous base stretch were designed. Each primer set was used to amplify the ambiguous base stretch by PCR using the following cycle; 95°C for 1 min followed by 35 cycles of 95°C for 30 s, varied annealing temperature dependent on T_m_ of the primer pair for 45 s, 72°C for 60 s followed by a final extension at 72°C for 5 min. To ensure each PCR product corresponded to the intended ambiguous bases location, 198 PCR products were individually sequenced bi-directionally and aligned against the CR1 draft genome (28 remaining contigs) to verify the location of each ambiguous base stretch and fill in the missing sequence. Interestingly, sequences of 10 ambiguous base stretches matched with the sequence of 10 small contigs (<1 kb in size), indicating these 10 small contigs actually fit within these 10 ambiguous base stretches. Thus, sequencing of ambiguous base stretches reduced the set of contigs from 28 to 18, where 10 small contigs merged into 10 ambiguous base stretches.

### Novel gap closure approach

**Figure 5:**
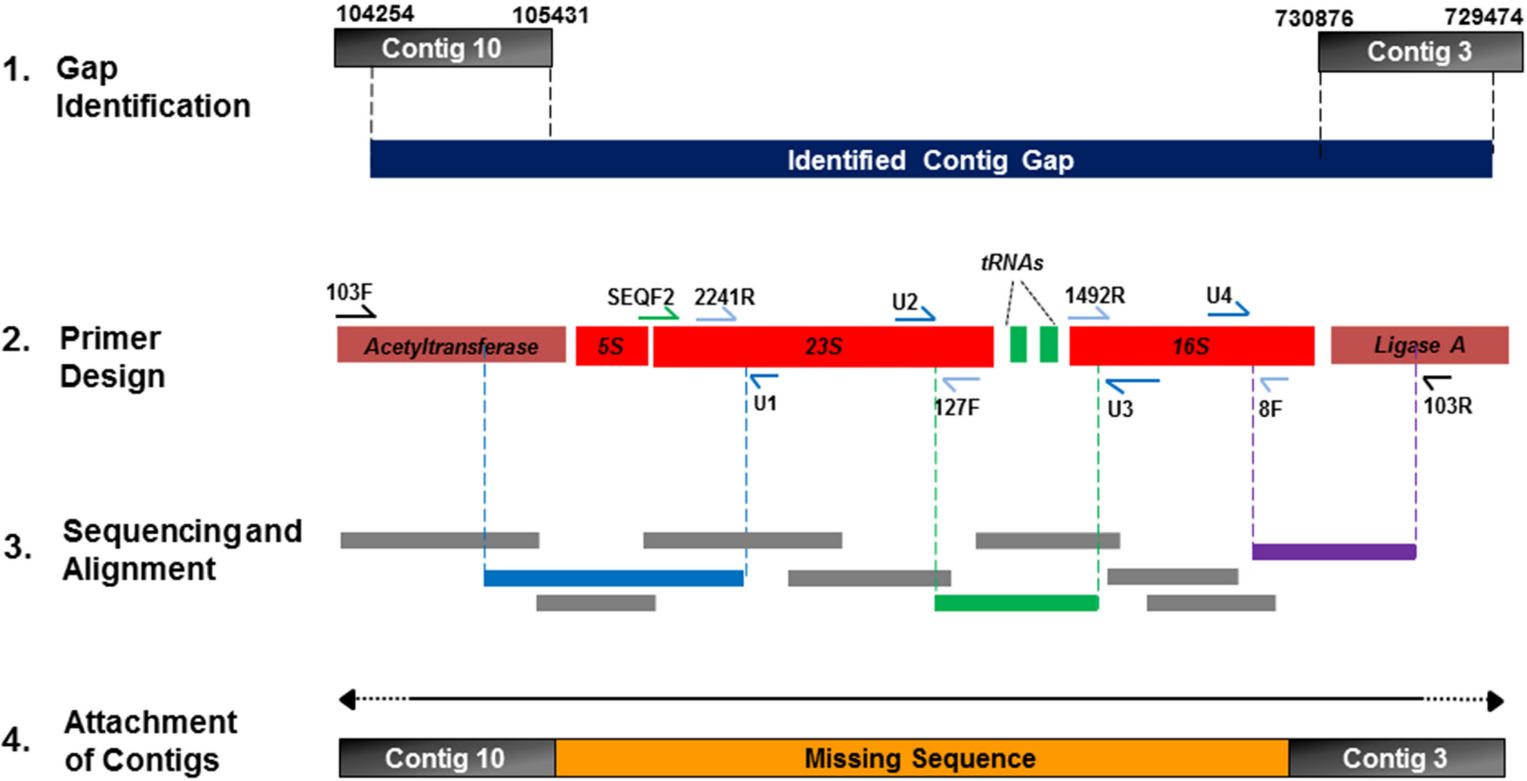
**Innovative primer walking strategy for sequencing of repetitive rRNA operons**. Putative gaps between contigs were identified by the contig-reordering tool within Mauve against other completely sequenced *P. polymyxa* strains. Primers U1, U2, U3, and U4 were designed to amplify the rRNA operon as depicted, allowing for rapid resolution of the rRNA gene flanking sequences and intragenic regions. Primers depicted were used as sequencing primers for Sanger sequencing of unknown regions and the resulting sequences were overlapped to identify missing sequence. Sequences of the depicted primers are presented in Table 1.

**Figure 6:**
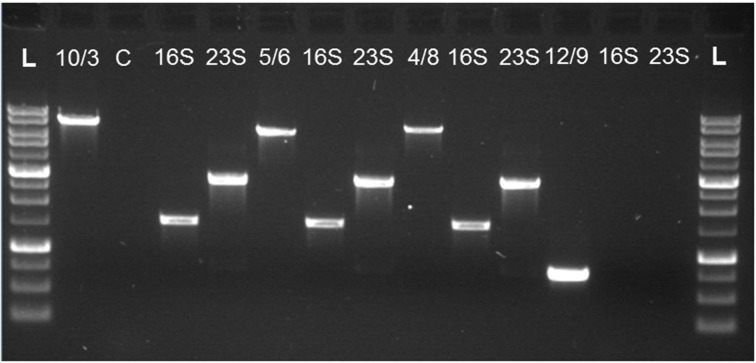
**Confirmation of the presence of rDNA operons located within contig gaps**. PCR products joining the ends of two contigs were used as templates for PCR to determine presence of rRNA genes within the gaps. Gaps that tested positive for rRNA operons were closed using our innovative rRNA gap sequencing procedure as illustrated in Figure 4. Gaps that did not contain rRNA operons required traditional primer walking to determine the missing sequence. PCR products labeled as 16S and 23S rRNA genes were amplified from the respective preceding PCR product corresponding to the following gaps; 10/3 – gap between contig 10 and contig 3, 5/6 – gap between contig 5 and contig 6, 8/4 – gap between contig 8 and contig 4, 12/9 – gap between contig 12 and contig 9. L represents a 1 kb molecular weight ladder and C is a representative template control utilizing distant primers not expected to be contained within the 10/3 gap to confirm template purity.

For gap closure of the CR1 draft genome, gaps (average length of 7.5 kb) between the 18 remaining contigs were identified through Mauve alignments against the complete genomes of *P. polymyxa* strain E681 and *P. terrae* strain HPL-003 (Figures 3, 4). Contig gaps were amplified from CR1 genomic DNA using oligonucleotides designed to hybridize between 250 and 350 bp from the 5′ and 3′ ends of each contig. PCR conditions for amplification of contig gaps were as follows; 95°C for 1 min followed by 40 cycles of 95°C for 30 s, varied annealing temperature dependent on T_m_ of the primer pair for 45 s, 72°C for 300 s followed with a final extension at 72°C for 20 min. All PCR amplifications of contig gaps were performed using Phusion^®^ Taq polymerase from NEB and products were excised and gel-purified. Absence of contaminating genomic DNA in gel-purified contig gap product was confirmed using primer sets corresponding to distant genomic DNA locations. During our masking of contigs comprised entirely of rDNA we noticed the prevalence of fragments of rDNA at contig boundaries. Since rRNA operons are multi-copy in any given bacterial genome, universal primers targeting 16S and 23S rRNA genes (Table 1) were used for PCR to determine if a candidate amplified gap (gel-purified PCR products as template) contained rDNA (Figures 5, 6). Through this method, we identified 12 out of the 18 remaining amplified contig gaps contained an rRNA operon. Their prevalence and localization to contig boundaries is likely a consequence of short read sequencing technology that cannot sequence across the entirety of an rRNA operon. For instance, the gaps between contigs 10 and 3 (10/3), contigs 5 and 6 (5/6), and contigs 8 and 4 (8/4) contain both 16S and 23S rDNA, while gap between contigs 12 and 9 (12/9) does not contain any rDNA (Figure 6). These results further confirmed our earlier assumption that the repetitive nature of rRNA operons was preventing a more complete *in silico* assembly.

Traditional primer walking involves Sanger sequencing using a primer complementary to the end of known sequence, resulting in approximately 750 bp to 1 kb of readable sequence per sequencing run. New primers are then designed to hybridize to the determined sequence and the process repeats itself until the gap is filled. To speed up gap closure, we designed several sets of rRNA gene specific primers (Primers U1, U2, U3, U4) that hybridize to the 5′ and 3′ end of both the 16S and 23S genes (Table 1) so that the entire rDNA operon contained in the contig gaps can be sequenced simultaneously including; 16S and 23S genes, the 16S/23S intragenic sequence, and gene flanking regions (Figure 5). Therefore, for each rDNA operon, six total sequencing reactions were performed in parallel (equivalent to three stages of primer walking). By taking advantage of the redundant nature of rDNA operons, 10 PCR amplified contig gaps containing rDNA operons were sequenced in parallel by this rDNA based primer walking approach using the designed rRNA gene specific primers (Table 1). In the majority of cases this strategy was sufficient to fill the contig gap in a single step, dramatically increasing the speed of the primer walking procedure. In cases where sequencing results did not reach 16S/23S genes or where the contig gap did not contain 16S/23S genes, traditional primer walking was necessary to fill the remaining gaps.

### Investigation of rearrangements

**Figure 7:**
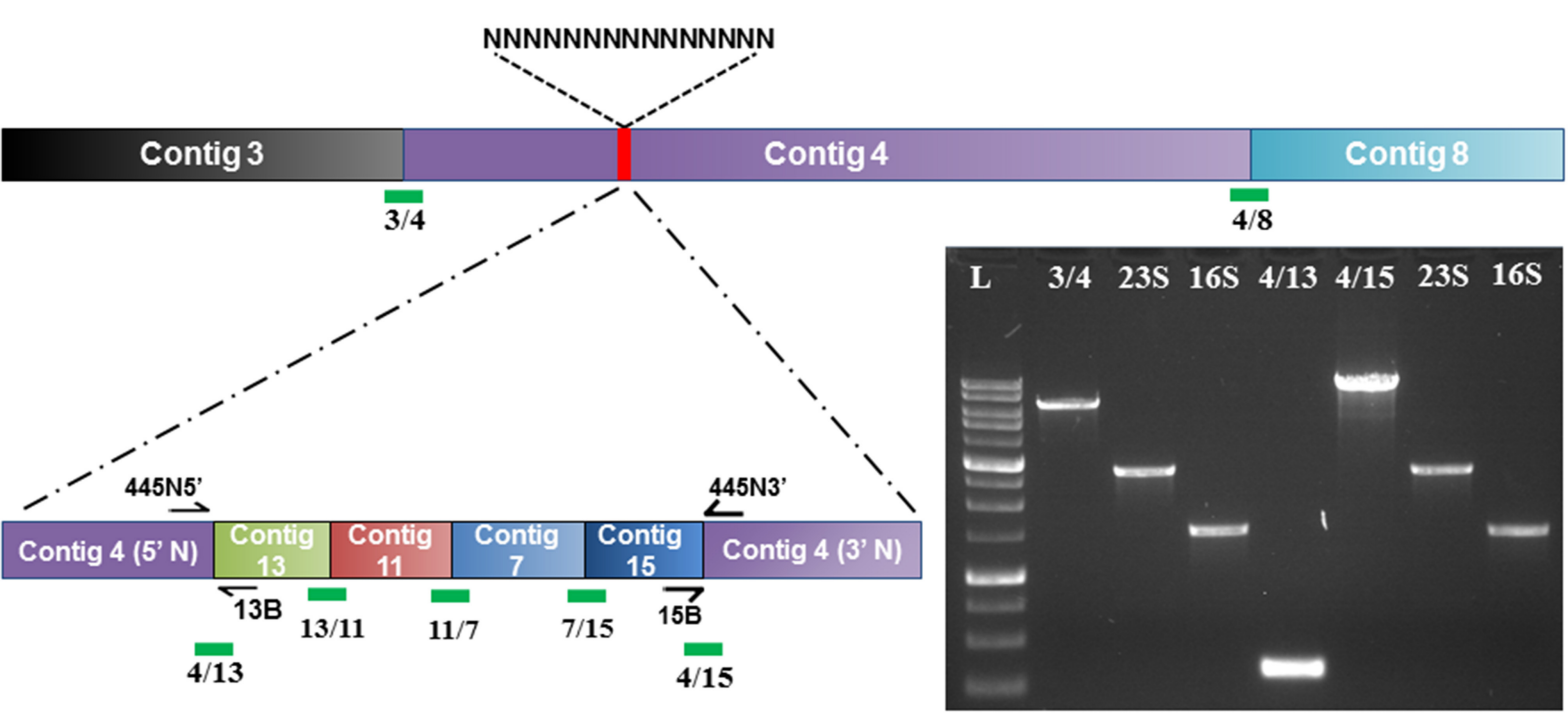
**Identification and resolution of rearrangement in draft contig assembly**. The combined contigs of 13, 11, 7, and 15 was predicted to be located within an ambiguous base stretch based on Mauve alignments, which was confirmed by PCR amplification of the adjoining ends of contigs 4(5′N) to contig 13 and contig 15 to contig 4 (3′N) as shown by lanes 4/13 and 4/15, respectively. L represents a 1 kb molecular weight ladder, 16S and 23S genes were amplified from the preceding PCR product correspond to the following contig gaps; 3/4 – gap between contig 3 and contig 4, 4/15 – PCR product corresponding to insertion of contig 15 within an ambiguous base stretch of contig 4, 4/13 – PCR product corresponding to insertion of contig 13 within an ambiguous base stretch of contig 4.

One of the most common errors present in draft genomes are rearrangements, a result of incorrect assignment of overlapping reads (Salzberg and Yorke, 2005). Typically, the PCR amplification of ambiguous base stretches during genome finishing is straightforward. For the CR1 genome, difficulties generating PCR product for an ambiguous base stretch located in contig 4 of the draft assembly suggested either an error in the scaffold assembly or a rearrangement in the draft genome. Analyzing low similarity regions in the Mauve alignments of the draft genome also indicated potential rearrangements in the CR1 draft genome assembly (compare local collinear block composition between CR1 and *P. polymyxa* strain E681, Figure 3). To pinpoint the location of the rearrangements, 10 kb windows of nucleotide sequence from the CR1 draft genome flanking the putative rearrangements were aligned using BLASTn against completely sequenced genome of *P. polymyxa* strain E681. Taken together, the results from Mauve alignments and BLASTn alignments suggested a large insertion of the assembled contigs of 11, 13, 7, and 15 immediately adjacent to an ambiguous base stretch in contig 4. Figure 7 offers a schematic representation of the final *P. polymyxa* strain CR1 genome assembly compared to the originally identified *in silico* assembly and scaffold. To verify the contig rearrangement, we designed the primers 13B and 445N5 to specifically amplify the joint region of contig 13 and the ambiguous base stretch in contig 4, as well as specific primers 15B and 445N3 that flanking the joint region of contig 15 and the ambiguous base stretch in contig 4 (Table 2). As expected, DNA sequences from the generated PCR products confirmed that the rearrangement present in contig 4 of the draft genome was an assembly error. The PCR product from primers 13B and 445N5, (gap between contig 4 and contig 13) was under 1 kb and was immediately filled by direct sequencing. Interestingly, the PCR product from primers 15B and 445N3 was quite large and contained both 16S and 23S rDNA and was verified and sequenced using the same rDNA based primer-walking strategy described above (Figure 7, lane 4/15). Resolution of the rearrangement uncovers an approximately 200 kb assembly error in the draft genome, and these types of large-scale rearrangements are commonly present in published draft genomes (Latreille et al., 2007).

**Table 2:** **Specific Primers targeting identified contig gaps for amplification**.

**Contig gap**	**Forward primer**	**Reverse primer**
5–1	AATAGACGGGTCTTCCTGCTTATAC	CGATACAACAGCCGACATTTGATTA
5–6	ACCCTAATCGACTGCTTAATCTTGT	CATCTATTGAACTCATTGAACGGGG
6–10	GATGAACCAAAACCTCACCAAGAAT	CGATTGGATCAAGATATTCGCTACG
10–3	GCATCCACAATACGACCCATAAAAT	GAATGTAGGACGAACGGGTAAAATC
3–4	CGGATTACCTCAAAAGGATTGGATG	AAAGAACCATCATGCACAGACAAAT
12–1	GTGCCGTTCTAATGTGTAGCTTATC	AGATGGATAGTAGTCAGGCAAATCC
9–2	GTATATCGGGGAAAGAGTAGGCAAT	GGTGTTTGTGTTGTAAGCTGTATGA
8–4	TTGTTTGATACGTTTGGTACCTTGG	TCTCCAAGTCAGCGTTCGTC
13–11	GACATGGTTGATTGAAAGTGACTGG	AGTGCTCAGCATGGAAGCAA
11–7	TGGTCAATGTAAAACGCAATCTTCA	CCATCATATCCGGGCACCAA
7–15	GCACTGAATAATCCCATTCTCAACC	TGAGGCAACAAGAATCCGCT
4–15	ACATGTTGCTTTCTTTTGCTGA	GGACAACCAGGATACCGCAA
4–13	AACCTGCTGATTATGCGGCT	AGTGCTTGCAAAGTTGGCTC

Each primer set was designed using Primer3 and tested for unintended priming using the Paenibacillus polymyxa strain CR1 draft genome as reference. Putative gaps were identified by Mauve alignments against closely related strains and species. Order of contigs listed does not imply orientation. Primer sets 4–15 and 4–13 were designed to confirm the identified rearrangement as described in Figure 7.

As a final validation of genome assembly, the sequence of the 38 contigs from the original draft assembly, including the contigs containing 16S and 23S rRNA gene fragments that were merged earlier, were aligned against the final contiguous assembly of the CR1 genome, which confirmed coverage of the entire draft in the final sequence.

### Genome annotation

The data contained in gene and protein annotations is ultimately the primary reasoning for genome sequencing. Automated *in silico* annotation suites rely on open reading frame predictions and homology searches against databases of characterized proteins. A multitude of automated annotation pipelines exist, each with their own respective algorithms, strengths, and limitations, and careful consideration must be taken when deciding which annotation suite to be used (Richardson and Watson, 2013). To provide consistency with other publically available genomes we utilized the NCBI Prokaryotic Genome Automatic Annotation Pipeline (PGAAP) for the annotation of the complete CR1 genome. The PGAAP uses a combination of Hidden Markov Model based gene prediction and a sequence similarity search against Entrez Protein Clusters, the Conserved Domain Database, and the Clusters of Orthologous Groups database. Annotations are automatically cross-referenced by homology to proteins contained in the NCBI database and are assigned standard locus tags, protein/gene names, and protein domain annotations. Annotation of the *P. polymyxa* strain CR1 genome using the PGAAP revealed 5283 protein-coding sequences, 36 rRNAs, 87 tRNAs and 103 pseudogenes. Despite the wealth of knowledge on structure-function relationships in prokaryotes, over 20% of the genes in the CR1 genome correspond to a COG category of general function prediction or unknown function (Eastman et al., 2014b).

## Discussion

**Figure 8:**
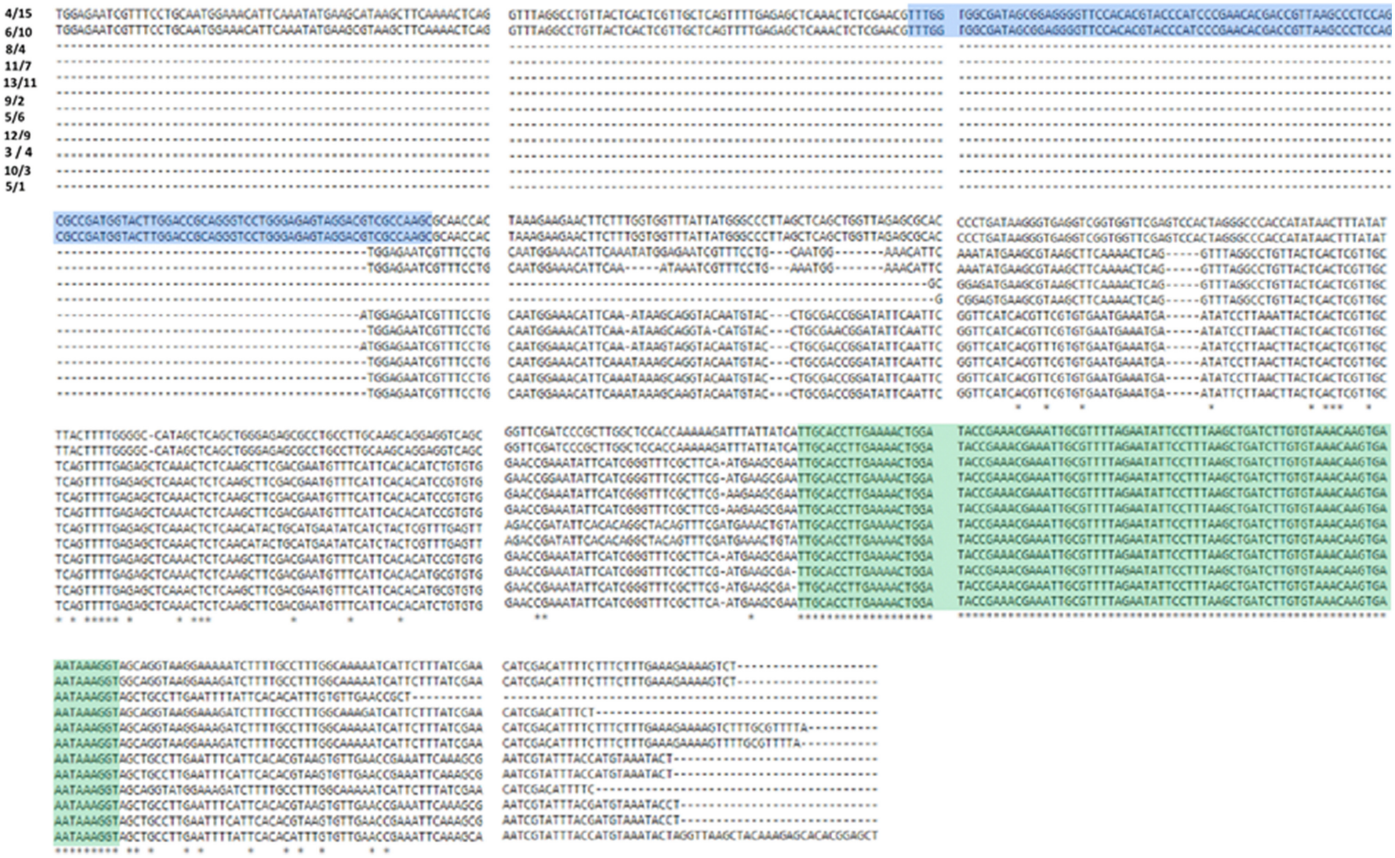
**ClustalΩ alignment of the 16S and 23S ribosomal subunit intragenic region of the CR1 genome**. Numbers to the left of sequence data represent the specific contig gap where the ribosomal subunit DNA was located. CR1 encoded ribosomal subunit DNA was unanimously localized to gaps from the draft genome. Intragenic DNA sequences were obtained using primers U2 and U3 and sequenced by Sanger sequencing and aligned by ClustalΩ using default parameters. Sequence highlighted in blue corresponds to 5S ribosomal subunit DNA, highlighted in green represents a highly conserved *P. polymyxa* species-specific 89 nt sequence.

**Figure 9:**
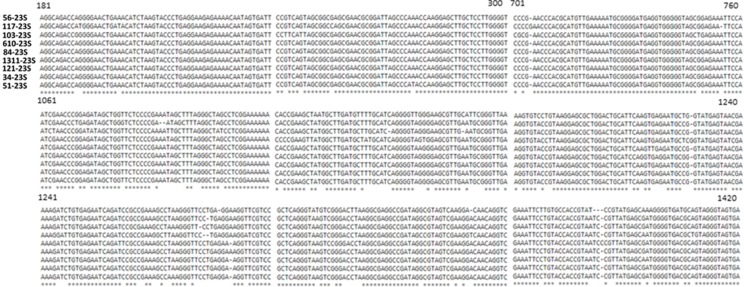
**ClustalΩ alignment of 23S ribosomal subunit regions from CR1 genome**. Numbers to the left of sequence data represent the specific contig gap where the ribosomal subunit DNA was located. Numbers above the nucleotide sequence data represent the position of each 60 nt frame. Regions not shown in the figure correspond to stretches of identical nucleotides between the depicted genes.

Regardless of the breakthroughs in sequencing technologies in the preceding decade and the power of second-generation sequencing platforms, generation of a complete, finished bacterial genome remains a difficult task. Draft genome contiguity is limited by the presence of repetitive genetic elements longer than short-insert and mate-pair read lengths, such as multiple copy and highly similar rDNA operons and tRNAs. Many sequencing and assembly errors inherent to second-generation sequencing technologies are present in draft assemblies as evidenced by a large number of ambiguous base stretches (Figure 4) and rearrangements (Figure 7) in the CR1 draft genome. The identification of rRNA gene fragments in a large proportion of contig ends in the draft CR1 genome reinforce previous findings that these types of repetitive genetic elements are a major limiting factor for *in silico* assembly (Rainey et al., 1996; Ricker et al., 2012). In addition, ribosomal subunit intragenic sequences are variable within an individual genome and it cannot be assumed that all rDNA operons have an identical composition (Figures 8, 9). Thus, the variety of features that need to be addressed during genome finishing requires a large amount of labor and resources to intensely scrutinize the draft genome sequence and experimentally confirm the correct assembly, arrangement, and structure.

The continued development and availability of simple and effective genome finishing strategies is paramount to accommodate the persistent onslaught of advances in sequencing technologies. Without concomitant advances in genome finishing strategies we cannot fully explore the nuances of evolution, phylogenetics, and prokaryotic genomic structure that can only be obtained from completed genomes.

As the costs of *de novo* genome sequencing continue to decrease, *de novo* genome sequencing will become increasingly common in the characterizations of bacterial strains. Based on the repetitive and conserved nature of rDNA operons we designed a procedure that allows for precise identification and sequencing of rDNA sequences within contig gaps. Using our procedure multiple primer walking sequencing reactions can be performed in parallel to target repetitive rDNA operons, thereby effectively reducing the time and resources needed for gap closure and assembly of scaffolded bacterial genomes. Despite recent developments in long read and single molecule sequencing technologies, due to the low base calling accuracy and high costs it is unlikely these technologies will completely replace Illumina sequencing for bacterial genome sequencing in the near future (Quail et al., 2012). Our approach increases the feasibility and speed of genome sequencing and offers an economical and technically simple strategy for microbial genome finishing, especially when available manpower and resources is a limiting factor in the decision to finish a genome.

### Nucleotide sequence accession numbers

The complete genomic sequences of strains referenced in the text are publically available in the NCBI GenBank database with the following accession numbers; *P. polymyxa* strain CR1 - NC_023037.1, *P. polymyxa* strain E681 - NC_014483.1, *P. terrae* strain HPL-003 - NC_016641.1.

## Author contributions

Alexander W. Eastman and Ze-Chun Yuan conceived and designed the study and drafted the manuscript. Alexander W. Eastman performed the data collection and analysis. Both authors approved the final manuscript.

### Conflict of interest statement

The authors declare that the research was conducted in the absence of any commercial or financial relationships that could be construed as a potential conflict of interest.
